# Partial hepatectomy for liver metastases from nasopharyngeal carcinoma: a comparative study and review of the literature

**DOI:** 10.1186/1471-2407-14-818

**Published:** 2014-11-07

**Authors:** Jun Huang, Qijiong Li, Yun Zheng, Jingxian Shen, Binkui Li, Ruhai Zou, Jianping Wang, Yunfei Yuan

**Affiliations:** Department of Hepatobiliary Oncology, Sun Yat-sen University Cancer Center, 651 Dongfeng Rd. E., Guangzhou, Guangdong 510060 China; Department of Surgery, The Sixth Affiliated Hospital of Sun Yat-sen University, Guangzhou, 510655 China; Department of Medical Imaging, Sun Yat-Sen University Cancer Center, Guangzhou, China; State Key Laboratory of Oncology in South China and Collaborative Innovation Center for Cancer Medicine, Sun Yat-Sen University, Guangzhou, China

**Keywords:** Nasopharyngeal carcinoma, Liver metastasis, Partial hepatectomy, Transarterial chemoembolization

## Abstract

**Background:**

The management of liver metastases from nasopharyngeal carcinoma (NPC) has not been extensively investigated. This study aimed to compare the long-term outcome of patients with liver metastases from NPC who were treated by a partial hepatectomy or transcatheter hepatic artery chemoembolization (TACE).

**Methods:**

Between January 1993 and December 2010, 830 patients were diagnosed with liver metastases from NPC and exhibited a complete response to the primary cancer of the nasopharynx and regional lymph nodes. Fifteen patients with intrahepatic metastasis underwent R0 partial hepatectomy. As a parallel control group, another 15 patients with a resectable liver metastasis who underwent TACE were selected. Prior to the resection and TACE that were performed on patients in these two groups, radical radiotherapy with or without adjuvant chemotherapy was administered. Clinicopathological data and treatment outcomes were compared retrospectively.

**Results:**

No significant differences were observed between the two groups in terms of the clinicopathological features, which include gender ratio, liver function, accompanying cirrhosis, rate of infection with the hepatitis B virus, tumor size, tumor number, pathological type and preoperative comorbidities. The 1-, 3- and 5-year overall survival rates from the time of hepatectomy were 85.7%, 64.2% and 40.2%, respectively, with a median survival of 45.2 months, whereas the 1-, 3- and 5-year overall survival rates were 53.3%, 26.6% and 20.0% for patients in the control group (P = 0.039), respectively, with a median survival of 14.1 months. The actuarial median progression-free survival (PFS) of the patients in the resection group was 21.2 months, and the 1-, 3- and 5-year PFS rates were 70%, 53% and 18%, respectively. In the control group, the 1-, 3- and 5-year PFS rates were 27%, 7% and 0.0% (P = 0.007), respectively, with a median survival of 4.2 months. Thus far, 5 patients have survived for more than 5 years, and the longest survival time is 168.1 months.

**Conclusions:**

For patients with limited liver metastases from NPC, hepatectomy provides a survival advantage over TACE. Due to the limited treatment options for patients with liver metastasis from NPC, hepatectomy should be recommended as an optimal treatment. Moreover, perioperative chemotherapy may be associated with an improved prognosis.

## Background

Nasopharyngeal carcinoma (NPC) is a common disease among Asians, especially the Southern Chinese. The incidence of the disease is 20–30 per 100,000 in Southeast Asia and less than 1 per 100,000 in western countries [1]. In contrast to other squamous cell carcinomas of the head and neck, NPC is characterized by a high tendency for metastatic dissemination [2]. The most common pathological type of NPC is non-keratinizing undifferentiated carcinoma (95% of patients in Southern China and 63% in North America), which can be managed with proper treatment, but has a high incidence of distant metastasis, compared to the other two types of NPC, squamous cell carcinoma and keratinizing undifferentiated carcinoma [1, 3]. NPC has a tendency to develop distant metastasis to the following organs: bones, lungs, liver, and distant lymph nodes, which is the main cause of death of patients with NPC [4]. The liver is one of the most common site of metastases from NPC; liver metastasis is usually multifocal with a worse prognosis than metastasis to the lung or bone [5]. Many patients with NPC who have liver metastasis are not eligible for a hepatectomy because of multiple lesions or the presence of extra-hepatic metastases. Although a high objective response rate has been obtained in some cases of metastatic NPC using chemoembolization and chemotherapy, these treatments are palliative and the patients’ overall survival rates are still not satisfactory [6–8].

The role of partial hepatectomy for hepatic metastasis from NPC is not well- documented. Hepatectomy for solitary colorectal liver metastasis is well-recognized as a standard treatment [9, 10]. However, very few studies have reported on the curative effect of hepatic resection for liver metastases from NPC. To our knowledge, the present study provides the largest consecutive series of patients treated by hepatectomy for liver metastases from NPC. This study aimed to identify the long-term outcome in patients with liver metastases from NPC who underwent hepatectomy and to compare the results with those who underwent TACE.

## Methods

### Patients

This retrospective study was approved by the ethics committee of Sun Yat-Sen University Cancer Center, and was in accordance with the Helsinki Declaration of 1975, as revised in 1983. Between January 1993 and December 2010, 830 consecutive patients with newly diagnosed liver metastases from NPC were treated in the Sun Yat-Sen University Cancer Center. NPC was histologically confirmed in all patients. The diagnosis of liver metastasis was based on histological evaluation, ultrasound and computed tomography of the abdomen. Patients with other malignant tumors were excluded. Among the 830 patients, 86 patients without extrahepatic metastases from NPC underwent partial hepatectomy or TACE. Of these cases, 15 patients who underwent partial hepatectomy were enrolled in the resection group. Of the 71 patients who underwent TACE, 15 patients with resectable hepatic metastatic lesions were treated with TACE because the patients refused to accept operation. These 15 patients were selected as the control group. Each group was composed of 12 males and 3 females, with a median age of 46 and 43 years in the resection group and the TACE group, respectively.

### Procedures

Computed tomography (CT) scans or magnetic resonance imaging (MRI) of the head and neck showed a complete response of all the primary carcinomas after radical radiotherapy with or without adjuvant chemotherapy. Emission computed tomography (ECT) for bones and CT for chest, or positron emission tomography-computed tomography (PET-CT) was performed to rule out extra-hepatic metastasis. Partial hepatectomy was performed only if no evidence of local recurrence or extrahepatic distant metastasis was observed, although there was no definite indication for such surgical treatment; nevertheless, rare cases were reported [11, 12]. Cardiopulmonary function and liver function, as determined by biochemical assays and Child–Pugh grading, of each patient were estimated. The criteria for resection were defined as follows: (1) complete response of primary disease to therapy, (2) no extrahepatic metastasis, and (3) solitary or multiple (no more than 5) liver metastases with at least two lesion-free segments. Partial hepatectomy was defined as the removal of the tumor plus a rim of non-neoplastic liver parenchyma, without regard to the anatomic segments as described by the Couinaud classification [13]. Major hepatectomy was defined as the resection of three or more hepatic segments according to Couinaud’s classification, and minor hepatectomy was defined as a resection of fewer than three hepatic segments [14, 15]. R0 resection was defined as a resection with a microscopically negative margin. An indocyanine green retention rate of 15 min (ICGR15) [16] was used to evaluate the liver function reserve. Intraoperative ultrasonography was also routinely used.

We used our previously reported protocol [17] for TACE, which was performed by the administration of 50 mg of epidoxorubicin, 300 mg of paraplatin and 6 mg of mitomycin, mixed 1:1 in an emulsion with lipiodol. The amount of lipiodol varied and was dependent on the tumor burden and vascular supply.

The treatments for tumor recurrence after hepatectomy included combinations of chemotherapy, radiofrequency ablation (RFA), percutaneous microwave ablation (PMA), and percutaneous ethanol injection (PEI). Hospital mortality was defined as death attributed to hepatectomy or TACE and all deaths that occurred during the same hospital admission.

The baseline data, including gender, age, hepatitis B virus infection, test for Epstein-Barr (EB)-related virus infection, liver function, synchronous or metachronous presentation of liver metastases with primary tumor, and comorbidities before hepatectomy, were collected and analyzed. In addition, the clinicopathological data and treatment results including radical or palliative resection, operative procedure, tumor burdens, postoperative complications, the interval from treatment to recurrence or metastasis, and the survival rates after treatment were considered. Overall survival (OS) was reported from the date of hepatectomy or TACE, while progression-free survival (PFS) was defined as the interval from the date of hepatectomy or TACE to the progression of the tumor, whether it was a local or a distant recurrence. Patients with any evidence of macroscopic lesions after hepatic resection were excluded from PFS analysis.

### Follow-up

The duration of follow-up was calculated from the day of hepatectomy or TACE to either the date of death or the last follow-up visit. The study was censored on May 30th, 2013. Follow-up imaging (contrast-enhanced CT or MRI) was performed after treatment. Further treatments were based on clinical evaluation, laboratory values, and imaging response. Patients with stable disease were imaged every 3–4 months. The follow-up visits consisted of a physical examination, routine blood tests, liver function tests, a determination of serum VCA-IgA and EA-IgA levels, an abdominal ultrasonography or computed tomography scan, a chest X-ray, and head and neck MRI. A bone scan or a positron emission tomography–computed tomography (PET-CT) scan was performed when there was evidence of local recurrence or distant metastasis.

### Statistics

The statistically significant differences in categorical and continuous numerical variables between the patients in the resection group and those in the control group were calculated using the Pearson chi-square test with Fisher’s exact test and the unpaired Student’s *t*-test, respectively. Overall survival rates and progression-free survival rates were analyzed by the Kaplan–Meier method, and the differences between the two groups were compared by the log-rank test. Alpha was set at 0.05, and all tests were two-tailed. All statistical analyses in this study were performed with the software package SPSS (Statistical Package for the Social Sciences) 17.0 (SPSS Inc., Chicago, IL).

## Results

The characteristics of the 30 patients are summarized in Table 1. The two groups were similar for all matching criteria, and no significant differences were found between the two groups with regards to demographics, tumor characteristics, primary treatments, preoperational comorbidities and postoperative complications. The median age was 46 years (range: 36–63 years) and 43 years (range: 26–63 years) for patients in the resection group and patients in the control group, respectively. Patients in the resection group had the same proportion of men and women as that of the control group, which was 12 men and 3 women (*P* > 0.999). There were no significant differences between the patients in the resection group and patients in the control group in terms of laboratory analyses, such as liver function and EB virus infection rates. Although we found 3 cases with hepatitis B virus infection, none of the patients suffered from severe cirrhosis. At the time of admission, the liver function grade for all patients was “A” according to the Child-Pugh grading system. The diagnoses were confirmed histologically for patients in the resection group. For patients in the control group, the 7 cases of metastases were confirmed histologically by a biopsy under the guidance of CT or ultrasound. The remaining 8 cases were clinically diagnosed by CT or magnetic resonance imaging with evidence of progressive enlargement of a hepatic lesion. The pathology type of all specimens was confirmed as undifferentiated non-keratinizing carcinoma. Only 1 patient in the control group demonstrated a synchronous NPC and liver metastasis, while the other 29 patients were found to have metachronous development of liver metastasis without any local recurrence. The number of liver lesions was less than 3 with diameter of 1.5 to 10 cm in 24 patients (24/30, 80%) and 3 to 4, with diameter of 1.5 to 7.0 cm in remaining 6 patients (6/30, 20%). Bone scans, PET-CT scans and chest X-rays or CT imaging showed no signs of bone, lung or other extrahepatic distant metastases.

**Table 1:** **Clinicopathological factors in 30 patients with liver metastases from Nasopharyngeal Carcinoma**

	Resection group	Control group	P value
	(n = 15)	(n = 15)	
Age (y)	46 (36–63)	43 (26–63)	>0.999
Gender			>0.999
Male	12 (80%)	12 (80%)	
Female	3 (20%)	3 (20%)	
Presentation			>0.999
Synchronous	0 (0%)	1 (6.7%)	
Metachronous	15 (100%)	14 (93.3%)	
Abdominal symptom			0.598
Positive	1 (6.7%)	3 (20%)	
Negative	14 (93.3%)	12 (80%)	
Interval from NPC to hepatic metastasis			0.427
<12 (mo)	3 (20%)	6 (40%)	
≥12 (mo)	12 (80%)	9 (60%)	
PFS (mo)			>0.999
<12 (mo)	7 (46.7%)	8 (53.3%)	
≥12 (mo)	8 (53.3%)	7 (46.7%)	
EBERs			>0.999
Positive	15 (100%)	15 (100%)	
Negative	0 (0%)	0 (0%)	
Child-Pugh Grading			>0.999
Grade A	15 (100%)	15 (100%)	
Grade B	0 (0%)	0 (0%)	
Hepatitis virus B infection			>0.999
Positive	2 (13.3%)	1 (6.7%)	
Negative	13 (86.7%)	14 (93.3%)	

Continuous data were expressed as median (range).

Categorical variables were reported with (%).

PFS: progression-free survival from diagnosis of NPC to discovery of liver metastasis.3.

EBERs: Epstein-Barr -Virus encoded small RNAs.

The treatments for the primary tumors in the two groups included radiotherapy and adjuvant chemotherapy. The median dose of radiation to treat the nasopharyngeal carcinomas was 70 Gray (range: 68 to 76 Gy) and 70 Gy (range: 66 to 78 Gy) in the resection group and the control group, respectively. For the regional lymph nodes, the median radiation dose was 66 Gy (range: 50 to 68 Gy) and 64 Gy (range: 50 to 70Gy) in the resection group and control group, respectively. No significant differences were found between the two groups (Table 2). The primary chemotherapy regimen used was fluorouracil (5-FU) combined with cisplatin (DDP) or other protocols based on these two agents. Additionally, there were no significant differences in the responses to adjuvant chemotherapies between the two groups (Table 2). The patient with synchronous liver metastasis underwent concurrent chemoradiotherapy for the primary tumor. All of the patients showed a complete response of the regional lymph nodes and nasopharyngeal mass after radiation therapy and chemotherapy.

**Table 2:** **Liver metastases-related characteristics in 30 patients with liver metastases from Nasopharyngeal Carcinoma**

	Resection group	Control group	P value
	(n = 15)	(n = 15)	
Lesion numbers			>0.999
<3	12 (80%)	11 (73.3%)	
≥3	3 (20%)	4 (26.7%)	
Size of largest metastasis (cm)			0.450
<5	8 (53.3%)	11 (73.3%)	
≥5	7 (46.7%)	4 (26.7%)	
Pathological type			>0.999
Undifferentiated	15 (100%)	15 (100%)	
Other types	0 (0%)	0 (0%)	
Dose of Primary Radiotherapy (Gy)			>0.999
Nasopharyngeal	70 (68–76)	70 (66–78)	
Regional lymph nodes	66 (50–68)	64 (50–70)	
Adjuvant chemotherapy cycles of NPC			0.390
<3	10 (66.7%)	13 (86.7%)	
≥3	5 (33.3%)	2 (13.3%)	
Pre-resection/TACE comorbidities			
Present	3 (20%)	4 (26.7%)	>0.999
Absent	12 (80%)	11 (73.3%)	
Post-resection/TACE comorbidities			0.651
Present	2 (13.3%)	4 (26.7%)	
Absent	13 (86.7%)	11 (73.3%)	
Hospital mortality	0 (0%)	0 (0%)	>0.999

Continuous data were expressed as median (range).

Categorical variables were reported with (%).

NPC: nasopharyngeal carcinoma.

TACE: trans-hepatic arterial chemoembolization.

The interval from the diagnosis of the primary disease to the identification of liver metastasis was similar between the two groups, with a median of 15.6 months for patients in the resection group (range: 1.8 to 74.5 months) versus 14.1 months for patients in the control group (range: 0.0 to 37.5 months). Moreover, there was no significant difference between the two groups in the PFS from the complete response of primary tumors to the occurrence of hepatic metastases. The median PFS was 12.6 months for patients in the resection group (range: 0.0 to 73.5 months) versus 11.9 months for patients in the control group (range: 0.0 to 34.0 months).

Of the 15 patients who underwent hepatic resection, no obvious hepatitis or ascites was found prior to surgery. Three patients underwent a major resection, and the others underwent a minor resection, including segmental resection and local resection. After specimen dissection, 26 lesions were found in patients in the resection group. Most of the metastatic masses were stiff with a clear border, except for 2 lesions with an obscure border. No capsule was found in the metastatic tumors. The median surgical margin was 2.0 cm (range: 0.3 to 4.0 cm). Each tumor specimen was carefully examined after removal from the patient, and we found that most of the hepatic NPC metastases displayed an infiltrating growth pattern with a deficiency of blood supply and lack of a capsule. Most of the lesions were isolated, and only two tumors demonstrated invasion of the adjacent organs and a thrombus.

We also observed a deficiency in blood supply in most of the hepatic NPC metastases in the TACE imaging. In the control group, 13 patients had tumors that were deficient in blood supply, and only 2 patients had tumors with a rich blood supply. As universally accepted in TACE, a deficient blood supply might weaken the effect of chemotherapy or embolization because chemotherapeutic agents depend on the blood supply to enter the tumor.

No perioperative deaths occurred in either group. Compared to patients in the control group, patients in the resection group showed no significant difference in the incidence of preoperative comorbidities (P = 1.000). One patient in the resection group exhibited postoperative hepatic insufficiency, while another developed hydrothorax. Post-TACE complications were found in 4 patients (26.7%, 4/15) in the control group.

The progression rate of the patients in the resection group was 73.3% (11/15), which was lower than that of the patients in the control group (100%, 15/15), but the difference was not statistically significant (*P* = 0.330). In the resection group, 7 patients developed only extra-hepatic metastases, and 1 patients developed only intra-hepatic recurrence and 3 patients had both intra- and extra-hepatic metastatic lesions after resection. This is in contrast to the control group, where 13 patients exhibited only intra-hepatic progression, and 2 patients had both intra- and extra-hepatic metastatic lesions. Multimodality therapies, including systemic chemotherapy, repeated resection, repeated TACE, PEI, PWA, RFA, radiotherapy, and biotherapy were used in the patients with progression. The types of post-resection/TACE treatments for the two groups were not significantly different (Table 3).

**Table 3:** **Treatments after progression for 30 patients underwent resection or transarterial chemoembolization for liver metastases from Nasopharyngeal Carcinoma**

	Resection group (n = 15)		Control group (n = 15)		P value
	n	%	n	%	
All treatments after hepatectomy/TACE	13		12		>0.999
Systemic Chemotherapy	9		7		0.715
Radiation (NP recurrence)	1		2		>0.999
Repeat resection	1		0		>0.999
Repeat transarterial chemoembolization	2		8		0.021
Radiofrequency therapy	2		3		>0.999
Percutaneous ethanol injection treatment	1		0		>0.999
Percutaneous microwave ablation therapy	1		1		>0.999
Biological therapy	1		0		>0.999
Hepatic arterial infusion	1		2		>0.999
Observation	2		3		>0.999

**Figure 1:**
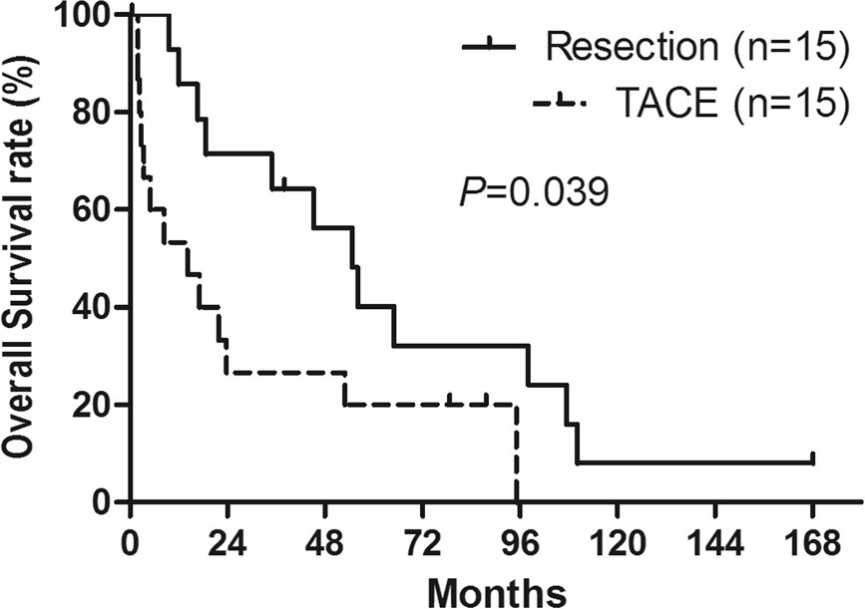
**The Kaplan–Meier survival analysis of the overall survival of the 15 patients with hepatic metastases from NPC who underwent resection and the 15 patients who underwent transhepatic arterial chemoembolization.**

**Figure 2:**
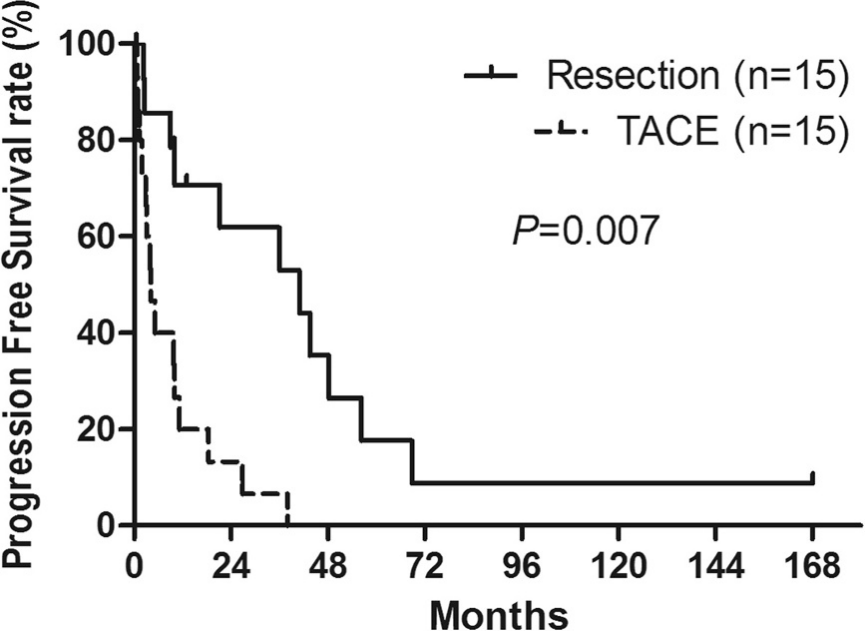
**The Kaplan–Meier survival analysis of the progression-free survival of the 15 patients with hepatic metastases from NPC who underwent resection and the 15 patients who underwent transhepatic arterial chemoembolization.**

A total of 11 patients in the resection group demonstrated a progression of the disease after partial hepatectomy, while 2 of the 4 patients without progression survived 168.1 and 13.0 months, respectively; the other 2 patients were censored because of lost follow-up. However, in the control group, 15 patients had tumor progression after TACE with one lost follow-up. The median OS time after hepatectomy was 45.2 months (range: 0.6 to 168.1 months), and the median OS time of the control group was 14.1 months (range: 2.1 to 95.2 months). Five patients in the resection group survived more than 5 years, including one 10-year survivor. The median PFS of the two groups was 21.2 months for patients in the resection group (range: 0.6 to 168.1 months) and 4.16 months for patients in the control group (range: 0.7 to 38.1 months). The OS rates for 1, 3, and 5 years after resection were 85.7%, 64.1%, and 40.2% for patients in the resection group, and 53.3%, 26.6%, and 20.0% for patients in the control group (Table 4). The postoperative long-term OS of the patients in the resection group was significantly better than that of patients in the control group (*P* = 0.039; Figure 1). When stratified by different resection methods, 11 patients underwent major resections with a median OS of 56.0 ± 13.2 months, and 4 patients with minor resections only had a median OS of 10.7 ± 3.7 months (*P* = 0.036). The PFS at 1, 3, and 5 years was 70.0%, 53.0%, and 18.0% for patients in the resection group and 27.0%, 7.0%, and 0.0% for patients in the control group (*P* = 0.007; Figure 2). The postoperative progression-free survival of patients in the resection group was significantly better than that of patients in the control group (*P* = 0.007; Figure 2).

**Table 4:** **Long-term outcomes of the 30 patients with liver metastases from Nasopharyngeal Carcinoma**

	Resection group	Control group	***P***Value
Median overall survival (mo)	45.2 ± 12.1	14.1 ± 8.6	0.039
1-y overall survival (%)	85.7	53.3	
3-y overall survival (%)	64.1	26.6	
5-y overall survival (%)	40.2	20.0	
Median progression-free survival (mo)	21.2 ± 11.0	4.16 ± 2.8	0.007
1-y progression-free survival (%)	70.0	27	
3-y progression-free survival (%)	53.0	7	
5-y progression-free survival (%)	18.0	0.0	
Progression (case)	11 (73.3%)	15 (100%)	0.330
Intra-hepatic	1 (6.7%)	13 (86.7%)	
Extra-hepatic	7 (46.7%)	0	
Both intra- and extra-hepatic	3 (20.0%)	2 (13.3%)

## Discussion

The main purpose of this study was to compare the outcomes of patients with NPC and liver metastasis who were treated with two different methods: partial hepatectomy and TACE. Our study showed that partial hepatectomy provided a survival advantage over TACE in patients with NPC and liver metastasis.

It has been reported that 30% to 60% of patients with locally advanced NPC will develop distant metastasis within 5 years, of which 5% to 8% present distant metastases at the time of diagnosis [2, 18]. The most common sites of metastasis are bones, followed by lungs, liver and distant lymph nodes. 78.3% patients occurred bone metastasis within 2 years after diagnosis of NPC, the overall survival time after bone metastasis is 6–24 months with a median of 12 months and the median survival time for patients who accepted alleviative treatment is merely 4 months [19]. Lung is the second common site of metastasis of NPC, patients with lung metastasis alone had a median overall survival of 3.9 years, which is significantly longer than that of other metastasis sites [20, 21]. Although liver has been reported to be the third most frequent site of NPC metastasis, with an incidence of 29.3% to 36% [22, 23], liver metastasis was the worst factor against prognosis: the median overall survival time after diagnosis of liver metastasis was only 3-5months [24]. Combination chemotherapy, which is usually palliative, is considered to be the standard treatment for metastatic NPC, especially for those patients with multiple metastases. The most common combination of cisplatin and 5-Fu was reported to generate a 66-76% response rate [25]. In the past 20 years, a number of patients with only intrahepatic metastases from NPC were experimentally treated with TACE as local chemotherapy combined with systemic chemotherapy. Despite the consensus regarding the chemosensitivity of NPC, there were only a few, sporadic reports that concerned the surgical treatment of patients with NPC and hepatic metastases after a complete response of the primary disease.

Due to recent advances in superior imaging techniques, more accurate preoperative exams, and improvements in the technical procedure of hepatectomy, hepatic resection of liver metastases has led to more curative results with fewer complications and lower mortality than in previous years [26, 27]. Hepatic resection has been reported to be an effective and potentially curative treatment for patients with liver metastases from colorectal and neuroendocrine carcinoma with a 5-year survival rate of 16%-76%, depending on the patients selected [28–30]. Compared to these cases, partial hepatectomy for NPC and hepatic metastases also provides a promising and inspiring outcome [12, 31]. In this study, to achieve satisfactory long-term outcomes, patients with hepatic metastases in both groups were strictly selected to avoid additional treatments for aggressive tumors with multiple metastatic sites. Patients who showed progression of extra-hepatic metastases after the diagnosis of liver metastases were not referred to hepatectomy. In addition, the patients in the control group were also well-matched to each patient in the resection group. The patients’ baseline characteristics were statistically identical in the two groups to avoid bias.

When the patients were stratified according to the method of hepatectomy, different survival results were obtained. In our study, compared to patients with a minor resection, the OS of the patients who underwent major resections was significantly better (*P* = 0.036). The comparison of outcomes between major and minor resection suggest a wider surgical margin for tumor resection may lead to better survival for the patients, which is supported by studies on liver metastases from colorectal cancer [26]. However, a major resection denotes less remnant liver, which is also the main paradox for hepatectomies for hepatocellular carcinoma (HCC). Unlike patients with HCC, patients with hepatic NPC metastases rarely have HBV infections, which lead to cirrhosis and a propensity for insufficient liver function after hepatectomy. Such characteristics of patients with metastatic NPC may allow for a greater resection of parenchyma. In our study, although 2 patients in the resection group developed postoperative hepatic insufficiency, both of them recovered within two weeks of hepatectomy; no post-treatment mortality was found in this study. However, more cases should be enrolled in our future study to further confirm the conclusion due to the limitated cases in this study.

TACE is regarded as a minimally invasive treatment protocol for liver metastasis [32]. It is also widely accepted as the appropriate treatment for advanced stage HCC [33] due to its limited damage to the liver and other organs that cause postoperative complications. In this study, patients in the resection group experienced more complications than patients in the control group (TACE), such as pain, fever, and severe hepatic insufficiency. However, after hepatectomy, none of the patients died of complications, or from sequelae after their discharge from the hospital. The treatments for progression of disease after hepatectomy or TACE were not significantly different between the two groups. We found 11 patients (73.3%, 11/15) in the resection group who experienced tumor recurrence after hepatectomy (37.0% at 1 year and 73.3% at 5 years) (Table 3). A total of 7 patients in the resection group developed only extra-hepatic metastases, and 1 patient developed only intra-hepatic recurrences and and 3 patients had both intra- and extra-hepatic metastatic lesions after resection. However, in the control group, 13 patients had only intra-hepatic progression, and 2 patients had both intra- and extra-hepatic metastases. The patients in the resection group had a lower progression rate than patients in the control group, although the difference was not statistically significant (*P* = 0.330).

It has been reported that the 1 year overall survival in patients with metastatic NPC ranged from 20 to 52% [2, 6, 18, 34]. However, in our study, the OS rates after hepatectomy at 1, 3, and 5 years were 85.7%, 64.1%, and 40.2%, respectively, for patients in the resection group. For the control group, the results were similar to previously reported values, and the OS rates at 1, 3, and 5 years were 53.3%, 26.6%, and 20.0%, respectively (Table 4). The postoperative long-term survival of the patients in the resection group was significantly better than that of the patients in the control group (*P* = 0.039; Figure 1) and that of patients from previous reports [2, 6]. Moreover, the patients in the resection group in our series had statistically better PFS rates than patients in the control group (*P* = 0.007; Figure 2). For primary or secondary carcinoma of the liver, hepatectomy is regarded as a curative treatment while TACE is regarded as a palliative treatment. The radical removal of the tumor decreases the chance for recurrence, while TACE leads to necrosis of the tumor by local chemotherapy and embolization. Another explanation for the poor PFS of TACE compared to hepatectomy is that TACE is based on a plentiful hepatic arterial blood supply, and thus the effects on the tumors were mostly dependent on this blood supply to the tumor. However, in our study, pre-procedure-enhanced CT or MRI and radiography of TACE showed that 13 of 15 patients had a poor blood supply to metastatic liver tumors, while only 2 patients had a rich blood supply. The poor blood supply impaired the expected effects on the tumor. In our study, the patient mortality was primarily cancer-related, and therefore the better PFS led to better OS for patients in the resection group than for patients in the control group. Furthermore, the disease-free interval between treatment of the primary tumor and the development of liver metastases is regarded as a surrogate marker of tumor biology. A longer PFS may indicate a less aggressive tumor; Teo and Ong reported that poor PFS (≤6 months) was a negative prognostic factor in metastatic NPC [35, 36].

So far, in this study, there were 7 patients in the resection group and 3 in the control group who survived more than 3 years after the end of the treatment. Among the 10 long-term survivors, 5 of the patients survived more than 5 years. The longest survivor has an overall survival time of 168.1 months without recurrence. Based on these results, we consider that patients in the resection group benefitted from hepatic resection. However, because the number of cases enrolled in our study was limited, we could not perform a multivariate analysis using the Cox proportional hazards model. Further research should be conducted to identify the prognostic factors of hepatectomy with a larger sample size.

The previously published long-term survival rates of patients with liver metastasis of NPC were also reviewed (Table 5). To our knowledge, this is the largest series of patients who were treated with resection for liver metastases from NPC. Among all 7 studies, we found 10 patients with hepatic metastases of NPC who were treated with chemotherapy. The longest overall survival time was 126 months, and the longest disease-free survival time was over 93 months. Only 3 patients had been treated by resection. The role of liver resection was controversial for patients with hepatic metastases due to a few studies with limited numbers.

**Table 5:** **Review of previously reports of long term survival of liver metastasis from Nasopharyngeal Carcinoma**

First author	Year	Case number	OS (mo)	PFS (mo)	Metastasis sites	Treatments for liver metastasis from Nasopharyngeal Carcinoma
Choo [37]	1991	1	36	36	Liver, bone	Cisplatin-based chemotherapy
Chung [38]	1997	1	126	42	Liver, lung, bone	5-Fu, leucovorin, ifosfamide
Fandi [6]	2000	2	>93	>93	Liver	Cisplatin-based chemotherapy
Ong [5]	2003	2	>60	NR	Liver	Cisplatin-based chemotherapy
Weitz [12]	2005	2	NR	NR	Liver	Hepatectomy (NR)
Delis [31]	2006	1	6	6	Liver	Segmentectomy
Khanfir [39]	2007	1	≥36	NR	Liver	Chemotherapy (NR)

NR: not reported.

## Conclusion

For the patients with resectable hepatic metastatic lesions, partial hepatectomy should be recommended after complete response of the primary tumor in the absence of extra-hepatic dissemination. In patients with resectable liver metastases from NPC, hepatic resection is safe and could be offered to acquire better long-term survival.
